# Initial HIV-1 viral load in French Guiana: Factors associated with viral load set point differences

**DOI:** 10.1016/j.ijregi.2024.100487

**Published:** 2024-11-10

**Authors:** Aude Lucarelli, Sébastien Rabier, Fanja Vergeade, Myriam El Guedj, Tania Vaz, Hawa Cisse, Loïc Epelboin, Philippe Abboud, Paul Le Turnier, Félix Djossou, Céline Michaud, Claudia Delin, Flavia Divino, Karine Verin, Ketty Bienvenu, Antoine Adenis, Mathieu Nacher

**Affiliations:** 1 COREVIH Guyane, Centre hospitalier de Cayenne, Cayenne, French Guiana; 2 Département de Santé Publique, Centre Hospitalier de l'Ouest Guyanais, Saint Laurent du Maroni, French Guiana; 3 Hopital de Jour Adultes, Centre hospitalier de Cayenne, Cayenne, French Guiana; 4 Service des maladies infectieuses et tropicales, Centre hospitalier de Cayenne, Cayenne, French Guiana; 5 Centres délocalisés de Prévention et de Soins, Centre hospitalier de Cayenne, Cayenne, French Guiana; 6 Centre Hospitalier de Kourou, Kourou, French Guiana; 7 CIC INSERM 1424, Centre Hospitalier de Cayenne, Cayenne, French Guiana; 8 Département Formation Recherche Santé, Université de Guyane, Cayenne, French Guiana

**Keywords:** HIV, Viral load set point, Sex, HIV subtype, Age, Transmission

## Abstract

**Objectives:**

HIV viral load set points may vary between virus subtypes and host characteristics. The HIV epidemic in French Guiana entails a mix of viruses and populations of cosmopolitan origins. In this epidemiological context, we aimed to determine whether, at the scale of our territory, we could identify differences in HIV-1 viral load setpoints in our hospital cohort.

**Methods:**

The French Guiana hospital cohort was retrospectively analyzed between 1992 and 2023. Bootstrapped (100 replications) quantile (median) regression modeled the initial HIV-1 viral load with age, sex, nationality, and cluster of differentiation (CD) 4 count at diagnosis as independent variables.

**Results:**

After adjusting for period of analysis and CD4 count at diagnosis, bootstrapped quantile regression (median) showed that Haitians (minus 20,088 copies, *P* <0.0001) and Guyanese (minus 14,087, *P* = 0.039) had a significantly lower viral load at HIV diagnosis than French, males had a greater viral load at HIV diagnosis than females (plus 16,430 copies, *P* <0.0001), each additional year of age was associated with more copies (+345 per year, *P* = 0.002). When compared to those with more than 500 CD4 per ml, persons with CD4 counts at diagnosis below 200 per ml had a greater initial viral load (plus 94,932 copies, *P* <0.0001), as did those with 200-350 CD4 per ml (plus 13,088 copies, *P* <0.0001), and those with (350-500 CD4 per ml) (plus 5643 copies, *P* <0.0001). Non-B viruses seemed to have an independently greater viral load at diagnosis than B viruses (plus 58,402, *P* = 0.029).

**Conclusions:**

We show some significant differences in the viral load set point of different groups. For nationalities, we are inclined to attribute this to differences in the frequency of infecting subtypes. This suggests that models of incidence or date of infection based on CD4 decline may be distorted by this situation.

## Highlights

• In French Guiana, three-quarters of HIV infections concern foreign persons.
• Viral load at diagnosis differed between sexes, nationalities, ages, and subtypes.
• This initial viral load reflects viral load setpoints of different HIV lineages.
• This questions the extrapolability of the cluster of differentiation 4 erosion modeling assumptions.

## Introduction

After an initial peak following infection, the HIV-1 viral load stabilizes and reaches its set point within a few weeks/months [1]. The set-point viral load correlates with a shorter time to advanced HIV [2] and increased infectiousness. In theory, there is thus a potential evolutionary trade-off represented by on the one hand greater virulence and probability of transmission, speedier immune suppression, vs lower virulence probability of transmission but perhaps a longer transmission potential. Hence, evolution may have optimized viral load setpoints to maximize transmission [3]. In practice, different studies have shown that sex—it is greater in males than in females—[4], human leukocyte antigen (HLA) B alleles and HLA A concordance between partners [5,6], HIV-1 subtype [7,8], and multiple infections with different HIV-1 subtypes [6] influence the viral load set point.

The French overseas territory of French Guiana lies in South America between Brazil and Suriname. The prevalence of HIV in French Guiana has been greater than 1% for over 3 decades. With the greatest GDP per capita in Latin America, it attracts numerous immigrants from HIV-endemic territories i.e., the Guiana Shield, the Caribbean, South America, and sub-Saharan Africa. In this context, over three-quarters of the HIV cohort are immigrants. However, we have shown, using two different methods [9,10], that over half of infections among foreigners were acquired after arrival in French Guiana, notably shortly after arrival when they are most vulnerable [11]. We have also shown that most infections concerned B subtypes, with 62.3% of B Caribbean HIV subtypes and 37.7% with B pandemic viruses [12]. With time, other HIV-1 subtypes and CRFs are increasingly encountered [13].

In this epidemiological context, we aimed to determine whether, at the scale of our territory, we could identify differences in HIV-1 viral load setpoints in our hospital cohort.

## Methods

### HIV care in French Guiana

French Guiana benefits from a universal health system with full coverage for all persons living with HIV for explorations and antiretroviral treatments. Undocumented foreigners may benefit from residence permits for health reasons. Outpatients are mainly followed in the three hospitals of Cayenne, Kourou, and Saint Laurent du Maroni.

### The French Guiana cohort

The French Guiana hospital cohort prospectively compiles data that was anonymized and retrospectively analyzed between 1992 and 2023. The variables studied were the first viral load measurement (before any antiretroviral treatment), age, sex, nationality, and the cluster of differentiation (CD) 4 count at diagnosis and virus subtype. Bootstrapped (100 replications) quantile (median) regression modeled the initial HIV-1 viral load with age, sex, nationality, and CD4 count at diagnosis as independent variables. The statistical significance level was 5%. The data was analyzed with STATA 16 (College Station, Texas, USA).

### Ethics and regulatory aspects

Patient records are entered in the eNADIS database and are merged with the French Hospital Database on HIV, ANRS CO4 cohort. The cohort has received regulatory approval by the Commission Nationale Informatique et Libertés (CNIL 2001/762876). As required by French law, all persons included in the cohort gave written consent for data collection and publication of research on the cohort.

## Results

Overall, there were 3169 patients with available initial HIV-1 viral load. There were 1592 females, 1576 males, and 6 female transgender patients included in the study. The mean age was 44.4 years (SD = 14.5). Males were older (mean = 46.1 years; SD = 14.8) than females (mean = 42.8 years [SD = 14], *P* <0.0001). Overall, 28% of patients were at the AIDS stage. The median CD4 at diagnosis was lower in males (256 per ml interquartile range [IQR] = 89-439) than in females (354 per ml IQR = 178-548), *P* <0.0001.

Among patients with available initial viral load, the main nationalities were: 1004 Haitians (31.6%), 613 (19.3%) French nationals, 541 (14.7%) Surinamese, 315 Brazilians (9.9%), and 219 Guyanese (6.9%). The median CD4 count at the time of HIV diagnosis was 314 per ml (IQR = 147-498) among Haitians, 368 per ml (IQR = 182-593) among French, 260 per ml (IQR = 102-443) among Surinamese, 225 per ml (IQR = 70-409) among Brazilians, and 357 per ml (IQR = 180-539) among Guyanese.

### HIV subtypes

Among patients with available HIV viral load at diagnosis 1627 (51.3%) had available HIV subtype data. Among the available subtypes over two-thirds were B (Table 1) and 27.7% were recombinant subtypes.

**Table 1:** HIV1 subtypes in the French Guiana cohort.

HIV-1 Subtype	N	%
B	1137	69.88
CRF08_BC	194	11.92
CRF28_BF	86	5.29
CRF012_BF	62	3.81
CRF29_BF	20	1.23
CRF02_AG	16	0.98
CRF03_AB	16	0.98
CRF17_BF	14	0.86
CRF07_BC	10	0.61
D	10	0.61
F	9	0.55
C	8	0.49
CRF01_AE	7	0.43
F1	6	0.37
CRF23_BG	4	0.25
CRF31_BC	4	0.25
CRF05_DF	3	0.18
CRF24_BG	3	0.18
A1	2	0.12
CRF014_BG	2	0.12
CRF40_BF	2	0.12
A2	1	0.06
CRF010_CD	1	0.06
CRF013_cpx	1	0.06
CRF015_01B	1	0.06
CRF016_A2D	1	0.06
CRF06_cpx	1	0.06
CRF18_cpx	1	0.06
CRF19_cpx	1	0.06
CRF30_06A1	1	0.06
F2	1	0.06
G	1	0.06
Untypable non-B	1	0.06

### Viral load at diagnosis

**Figure 1:**
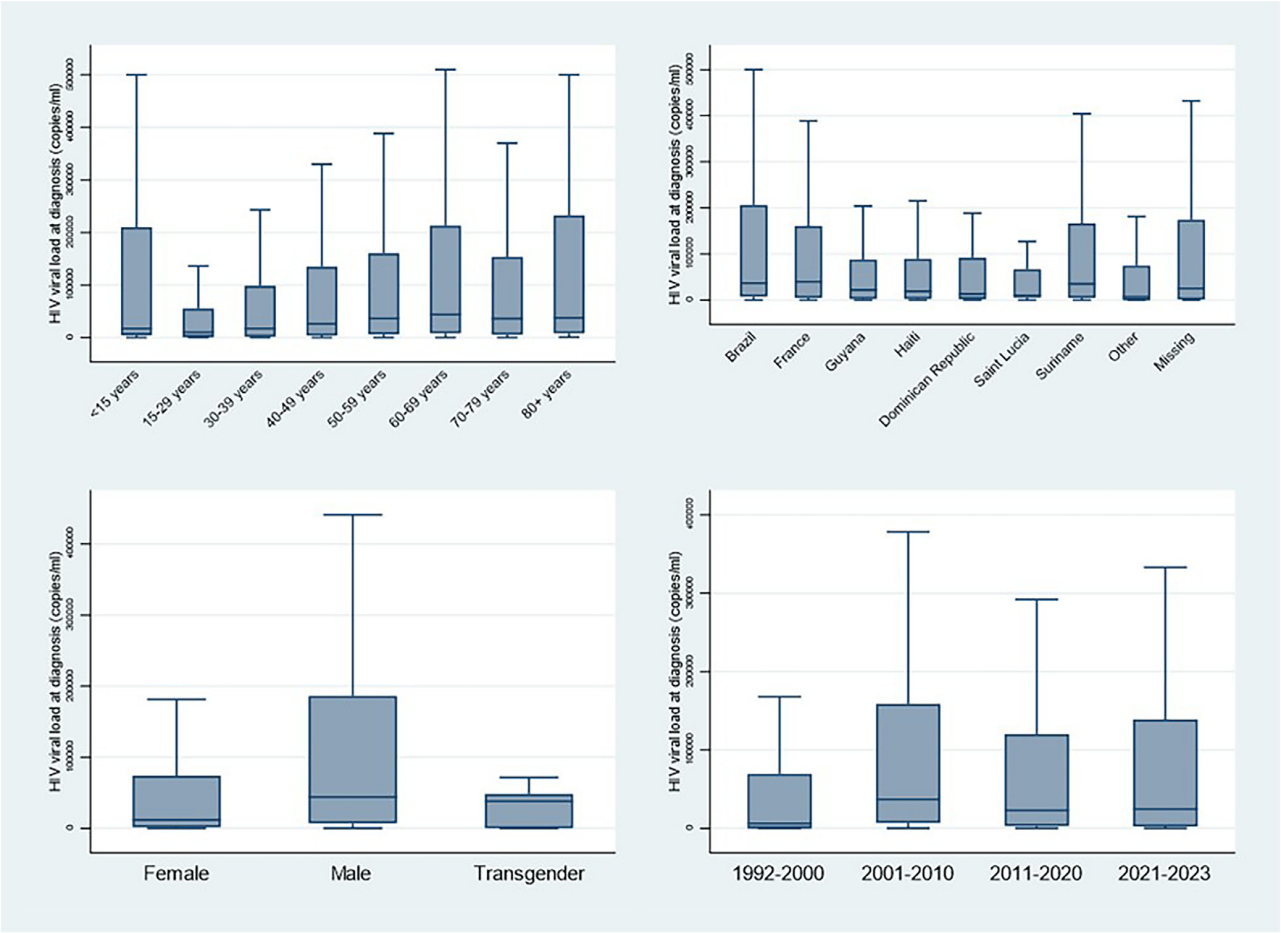
Initial viral load at HIV diagnosis by different categories, French Guiana, 1992-2023.

The median viral load at diagnosis was 24,000 copies per ml (IQR = 3065-120 431). Figure 1 shows that viral load at diagnosis was different between sexes, age groups, nationalities, and time periods. Hence males had greater viral loads than females, apart from the exception of those younger than 15 years, older age groups tended to have greater viral loads, those from Haiti, the Dominican Republic, Guyana, and Saint Lucia seemed to have lower initial viral loads than French or Brazilians, and viral loads before 2000 seemed lower than after 2000.

**Figure 2:**
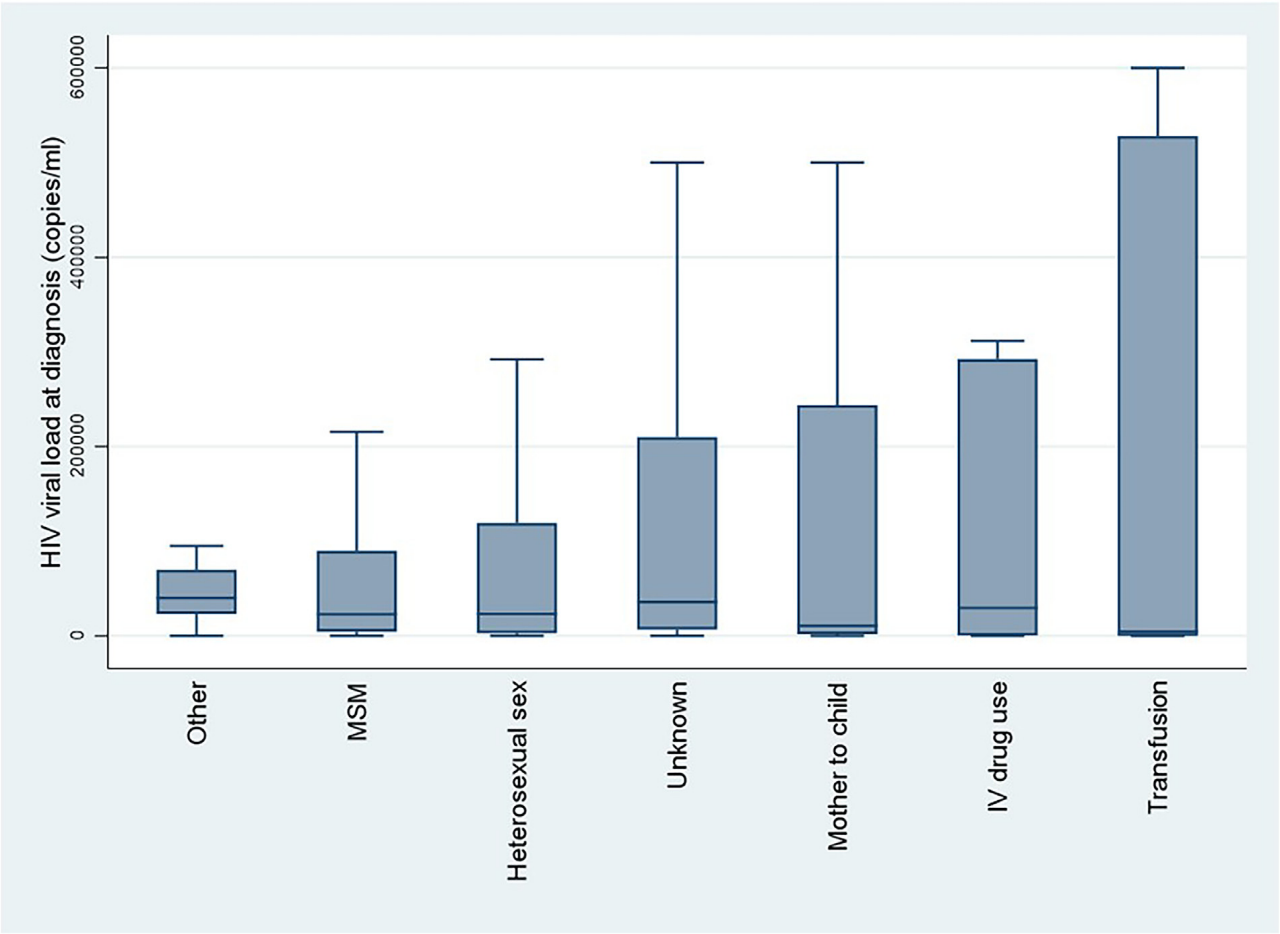
Initial HIV-1 viral load by transmission mode, French Guiana, 1992-2023.

The initial viral load at diagnosis was highest for parenteral transmission (intravenous drug use or transfusion) and for mother-to-child transmission (Figure 2).

**Figure 3:**
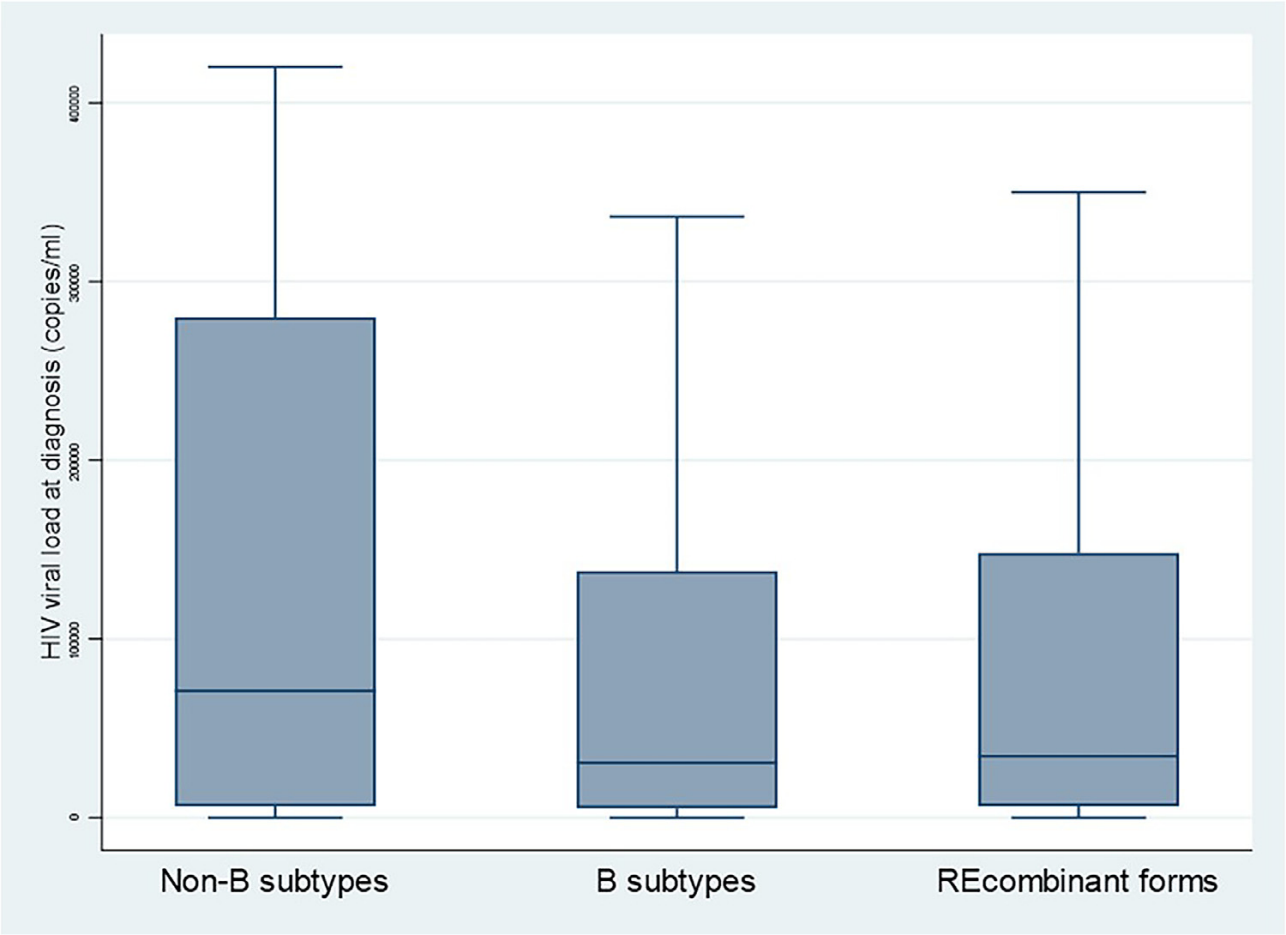
Initial HIV-1 viral load at diagnosis by subtype, French Guiana, 1992-2023.

Because the number of virus subtypes was large with most categories with few individuals we categorized the variable as B, non-B, and recombinant forms. Figure 3 shows that non-B subtypes seemed to have a greater initial viral load, but given the small numbers, statistical analyses were underpowered (24 transfusions, 37 IV drug users, and 132 mother-to-child transmissions).

### Multivariate analysis

After adjusting for period of analysis and CD4 count at diagnosis, bootstrapped quantile regression (median) showed that Haitians (minus 20,088 copies, *P* <0.0001) and Guyanese (minus 14,087, *P* = 0.039) had a significantly lower viral load at HIV diagnosis than French, males had a greater viral load at HIV diagnosis than females (plus 16,430 copies, *P* <0.0001), each additional year of age was associated with more copies (+345 per year, *P* = 0.002). When compared to those with more than 500 CD4 per ml, persons with CD4 counts at diagnosis below 200 per ml had a greater initial viral load (plus 94,932 copies, *P* <0.0001), as did those with (200-350 CD4 per ml) (plus 13,088 copies, *P* <0.0001), those with (350-500 CD4 per ml) (plus 5643 copies, *P* <0.0001). However, after adjustments, there was no difference between different time periods, so the variable was removed from the model. A smaller quantile regression model with only persons with available subtypes, recoding the variable in B, non-B, and recombinant found that non-B viruses seemed to have an independently greater viral load at diagnosis than B viruses (plus 58,402, *P* = 0.029).

## Discussion

Here we showed that there were significant differences between groups regarding the viral load at the time of diagnosis in French Guiana. Hence patients from Haiti and Guyana had lower viral loads at the time of diagnosis than French patients. As shown elsewhere males had greater initial viral loads than females. Those with lower initial CD4 counts had greater initial viral loads but it is worth pointing out that persons from Haiti had lower CD4 counts than French people, yet they also had lower viral loads. Among adults, there was a gradual increase in initial viral load with age. However, among those aged less than 15, mostly infected through mother-to-child transmission, the initial viral load seemed greater than among adults. However, there were only 55 such patients, and verification through adjusted analyses was impossible. Similarly, statistical comparisons between modes of transmission were impossible given the small numbers of parenteral transmissions in our cohort. Finally, non-B subtypes seemed to have greater initial viral loads than B subtypes.

Given the relatively short time to reach viral load set points and given the fact that estimated intervals between infection and diagnosis in French Guiana oscillate around 4 years [14], we consider that the viral load at the time of diagnosis represents the set point viral load.

In the cosmopolitan context of French Guiana, the above differences between populations could be hypothesized to reflect differences in the main virus subtypes. Hence, we have shown that for subtype B viruses in our cohort, there is a preponderance of B_Car_ over B Pandemic isolates. This reflects the deep historical and continuous connection with the Caribbean epidemic. However, since sexual transmission occurs at least partly within populations of the same nationality, it is likely that the proportion of different virus subtypes varies by nationality. More specifically, among Haitians we could hypothesize that B Caribbean subtypes would be far more frequent than among French nationals who may harbor these genotypes but seem also more likely to encounter B Pandemic subtypes that are preponderant in mainland France. Indeed, on a much smaller sample, we have shown that viral loads were lower among those with B Caribbean subtypes than among B Pandemic subtypes [12]. By extension, persons from Guyana, the Dominican Republic may share this preponderance of B Caribbean subtypes.

As others have shown, apart from HIV-1 subtypes, host factors such as HLA alleles can influence viral load set points, and it is thus conceivable that some alleles may be more frequent in some populations [5]. Sex has also been shown to affect viral load set points [4]. To our knowledge, the observation that viral load set point is proportional to age in adults has not been reported elsewhere. This association could reflect the influence of a gradual age-related immunological decline regarding the control of viral replication.

The present study has several limitations. First, we can only speculate on what lies behind the statistical associations. We did not conduct any analysis of the polymerase sequences to differentiate B subtypes in B Caribbean or B Pandemic or we do not have HLA allele analysis. The statistical analyses were often impossible when categories were too small (IV drug users, transfusion, mother-to-child transmission, rare virus subtypes). Nevertheless, even if today's testing efforts and immediate antiretroviral treatment avoid much of the erosion of the patient's immunity, the present results shed an interesting light on our epidemic. We have tried to estimate intervals between infection and diagnosis, and the proportions of foreign persons infected after their arrival in French Guiana based on CD4 erosion models. These estimates are useful, but the present results suggest that CD4 erosion rates may vary between population groups leading to distortions in the estimated durations.

In conclusion, we show some significant differences in the viral load set point of different groups. For nationalities, we are inclined to attribute this to differences in the frequency of infecting subtypes. This suggests that models of incidence or date of infection based on CD4 decline may be distorted by this situation.

## Declarations of competing interest

The authors have no competing interests to declare.

## References

[1] Huang X, Chen H, Li W, Li H, Jin X, Perelson AS (2012). Precise determination of time to reach viral load set point after acute HIV-1 infection. J Acquir Immune Defic Syndr.

[2] Lavreys L, Baeten JM, Chohan V, McClelland RS, Hassan WM, Richardson BA (2006). Higher set point plasma viral load and more-severe acute HIV Type 1 (HIV-1) illness predict mortality among high-risk HIV-1–infected African women. Clin Infect Dis.

[3] Fraser C, Hollingsworth TD, Chapman R, de Wolf F, Hanage WP. (2007). Variation in HIV-1 set-point viral load: epidemiological analysis and an evolutionary hypothesis. Proc Natl Acad Sci U S A.

[4] Sterling TR, Vlahov D, Astemborski J, Hoover DR, Margolick JB, Quinn TC. (2001). Initial plasma HIV-1 RNA levels and progression to AIDS in women and men. N Engl J Med.

[5] Mackelprang RD, Carrington M, Thomas KK, Hughes JP, Baeten JM, Wald A (2015). Host genetic and viral determinants of HIV-1 RNA set point among HIV-1 seroconverters from sub-Saharan Africa. J Virol.

[6] Saathoff E, Pritsch M, Geldmacher C, Hoffmann O, Koehler RN, Maboko L (2010). Viral and host factors associated with the HIV-1 viral load setpoint in adults from Mbeya region, Tanzania. J Acquir Immune Defic Syndr.

[7] Hollingsworth TD, Laeyendecker O, Shirreff G, Donnelly CA, Serwadda D, Wawer MJ (2010). HIV-1 transmitting couples have similar viral load set-points in Rakai, Uganda. PLoS Pathog.

[8] Bello G, Arantes I, Lacoste V, Ouka M, Boncy J, Césaire R (2019). Phylogeographic analyses reveal the early expansion and frequent bidirectional cross-border transmissions of non-pandemic HIV-1 Subtype B strains in Hispaniola. Front Microbiol.

[9] Nacher M, Adriouch L, Van Melle A, Parriault M-C, Adenis A, Couppié P. (2018). Country of infection among HIV-infected patients born abroad living in French Guiana. PLoS One.

[10] Arantes I, Bello G, Darcissac E, Lacoste V, Nacher M. (2021). Using phylogenetic surveillance and epidemiological data to understand the HIV-1 transmission dynamics in French Guiana. AIDS.

[11] Nacher M, Lucarelli A, Huber F, Rabier S, Douine M, Adenis A (2022). HIV-infection among immigrants in French Guiana: high risk during the first years after arrival. Cadernos de S aúde.

[12] Bello G, Nacher M, Divino F, Darcissac E, Mir D, Lacoste V. (2018). The HIV-1 Subtype B epidemic in French Guiana and Suriname is driven by ongoing transmissions of pandemic and non-pandemic lineages. Front Microbiol.

[13] Bello G, Delatorre E, Lacoste V, Darcissac E, Herrmann-Storck C, Tressières B (2020). Increasing prevalence and local transmission of non-B HIV-1 subtypes in the French Antilles and French Guiana between 1995 and 2018. Virus Evol.

[14] Nacher M, Adenis A, Huber F, Hallet E, Abboud P, Mosnier E (2018). Estimation of the duration between HIV seroconversion and HIV diagnosis in different population groups in French Guiana: strategic information to reduce the proportion of undiagnosed infections. PLoS One.

